# Dengue outbreaks in South Asia amid Covid-19: Epidemiology, transmission, and mitigation strategies

**DOI:** 10.3389/fpubh.2022.1060043

**Published:** 2022-12-15

**Authors:** Subhanwita Manna, Prakasini Satapathy, Ishani Bora, Bijaya Kumar Padhi

**Affiliations:** ^1^Department of Public Health, Indian Institute of Public Health, New Delhi, India; ^2^Department of Virology, Postgraduate Institute of Medical Education and Research, Chandigarh, India; ^3^Department of Community Medicine and School of Public Health, Postgraduate Institute of Medical Education and Research, Chandigarh, India

**Keywords:** dengue (DENV), South Asia, EPI-epidemiology, vaccine, transmission, mitigation strategies

## Introduction

The COVID-19 pandemic has affected people worldwide for the past 3 years and is now a precursor to several vector-borne outbreaks in low- and middle-income countries. South Asia is one of the most affected regions in the world due to the rise in COVID-19 cases and the disease's unregulated transmission. The World Health Organization (WHO) reported a 6.2% increase in COVID-19 incidence vs. other regions, which had a 5% drop (1). Although there is an increasing trend of COVID-19 infection in, this region has a long history of vector-borne diseases, particularly mosquito-borne diseases such as Dengue, Malaria, and others, due to its favorable environment. The high rainfall, high humidity, and temperature make it an ideal ground for mosquitoes to breed and grow during the summer and fall seasons. Despite its long history, the dengue virus remains one of the most pressing public health issues in the region.

Dengue fever is a vector-born viral disease transmitted by female mosquitoes, mostly *Aedes aegypti* and, to a lesser extent, *Aedes albopictus*, which are also carriers of yellow fever, Chikungunya, and Zika viruses. Before 1970, major dengue epidemics erupted only in nine countries. With Asia accounting for 70% of the worldwide disease burden, the disease is endemic in more than 100 nations (2). Dengue cases have increased significantly throughout the world in recent decades. Due to the fact that many dengue cases are mild, asymptomatic, and self-managed, the disease is underreported. Most often, other febrile diseases are misdiagnosed (3). There were more cases of dengue in 2019 than in previous years. Afghanistan witnessed its first appearance of dengue transmission, and all regions were affected. Several Southeast Asian countries were affected by dengue in 2022 (2).

## Post-COVID-19 dengue outbreaks in South Asian countries

**Figure 1:**
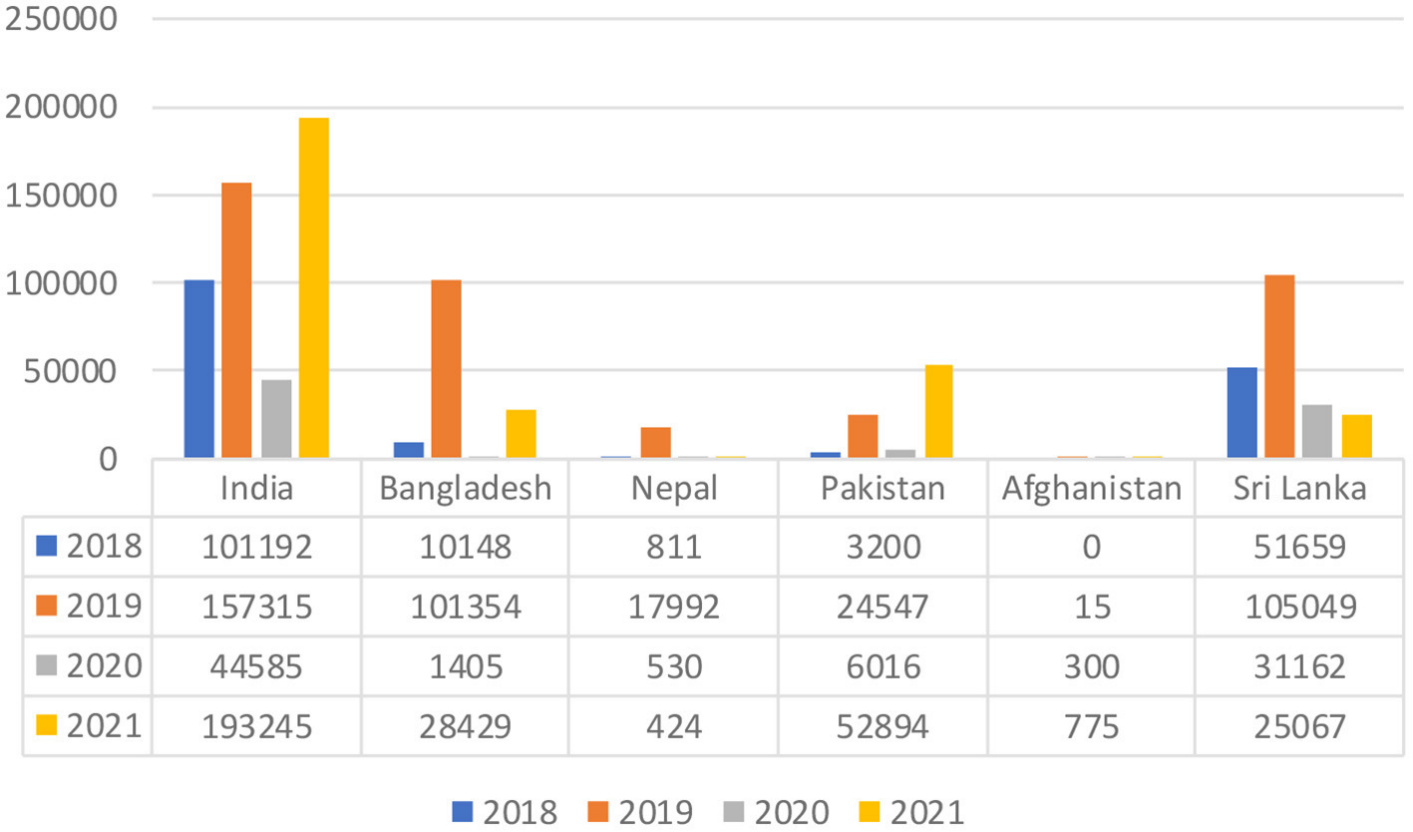
Trend of dengue-confirmed cases in South Asian countries. Data Source: We have extracted the confirmed Dengue cases as reported by the official websites of the above countries (4–9).

The ongoing pandemic poses a significant threat to the health system of all countries around the world. The increasing pandemic of COVID-19 poses a challenge to authorities of these countries, who have worked over the years to reduce the incidence of dengue. As a result, the COVID-19 pandemic has seen an increase in the number of dengue cases, which is still significant. Compared to the number of cases registered in 2020, in India, the prevalence of dengue fever has more than tripled (44,585 vs. 193,245 cases). In Pakistan, it climbed more than seven times (6,016 vs. 52,894 cases), in Bangladesh it recorded an increase of more than 19 times (1,405 vs. 28,429) in 2021 (Figure 1).

Although lockdown and social limitations would lessen the burden of dengue (10), it has been surprising to see that the caseload has significantly in South Asian countries despite the rigorous restrictions put in place by these countries from early 2021. However, the reported cases frequently go undiagnosed because they are asymptomatic or have unusual symptoms compared to a typical case of dengue fever. Furthermore, rapid progression and severe consequences such as cardiac and neurological symptoms, particularly in children, suggest a fatal coinfection with SARS-CoV2 in this region (11). Recurrent dengue infection is widespread in these nations, making it more severe due to the onset of a phenomenon known as antibody-dependent enhancement (ADE) (12), which is also observed in cases of recurrent COVID infections, making the coexistence of both diseases more lethal.

The restrictions of the COVID-19 pandemic are lifted worldwide in 2022, but in the interim, Southeast Asia had to deal with the highly contagious variation of the Omicron. On May 31, 2022, the data shows that 10,172 cases and three deaths had been reported in India. As of 21 August, 695 cases had been documented, with no deaths in the Maldives, representing an upsurge of 463 cases since 24th July 2022. On August 20, 1,807 cases had been registered, with 875 confirmed cases and no deaths in Pakistan, with an increase of 932 cases from July 4, 2022. On July 24, 2022, there had been a total of 7,687 cases and six fatalities reported, representing 93% of the total number of cases reported since the upsurge of cases in Bangladesh at the end of May 2022 (13). Recent news reports from various nations indicated an increase in dengue cases across the country in this region (14–16).

India has an interesting pattern for dengue outbreaks. The burden of the case rotates to southern India every 5 years. 60% of the overall caseload in 2022 came from Andhra Pradesh, Tamil Nadu, and Karnataka; however, a similar trend was observed in 2012 and 2017. Dengue fever spreads with the arrival of the monsoon season in the southwest and northeastern parts of India, with Maharashtra and Kerala accounting for the lion's share of dengue-related deaths.

## Epidemiology

The dengue virus (DENV) is a single-strand positive-sense RNA virus, a member of the Flaviviridae family of viruses. A viral genome and C proteins form the spherical shape. The nucleoplasmid, the viral core, consists of both of these parts. The viral envelope, which is a lipid bilayer membrane, also includes a group of proteins called E and M proteins. This arthropod-borne disease is caused by one or more of the four dengue viruses (DENV1–4). These four classifications are referred to as Siri types due to their propensity to interact in various ways with the antibodies present in the host serum. The names of the serotypes were determined by finding order. In 1943, in Japan, Ren Kimura and Susumu Hotta discovered the original identification of serotype 1 (17). Approximately 65% of the genomes of each of these serotypes are shared, while variances in the remaining 25% of their genomes have led to the emergence of different genotypes within serotypes (18). A sample of DENV-5 was found in a 37-year-old patient who had been admitted to the Sarawak state of Malaysia in 2007; the discovery was made in 2013, however, this group has not been reported since then (19). DENV strains continued to expand around the world while maintaining a high degree of genetic similarity within the region.

## Transmission

The transmission of DENV is mainly horizontal through the bite of an infected female mosquito of the Aedes genus, namely *A. aegypti* or *A. albopictus* (16). These mosquitoes get infected after ingestion of blood, form a host with viremia following which the virus stays in the midgut as the viral genome is stable there and later the virus disseminates in the secondary tissue which includes the salivary glands from where the virions can be released (17). This time between this ingestion of blood from the host to successive transmission of virus through different host through its bite is called the extrinsic incubation period and its usually 7–14 days (17). Moreover, it has also been observed that some of the arbovirus has the ability to vertically transfer the infection to their offspring during oviposition or within the ovary. This phenomenon plays an important role in maintaining the circulation of these viruses within the mosquito population (16).

## Mitigation strategies

Dengue virus control must be long-lasting and sustainable to control future outbreaks. The primary technique to avoid dengue fever is vector control, which uses chemicals such as insecticides, adulticides, and chemical compounds to keep water sources clean. This method is in addition to the challenging effort of producing a vaccine. Insect growth regulators (IGRs) are the most widely used chemical agent used to prevent the early stages of insect growth and development. Many IGRs, where dengue is reemerging, have shown resistance to the organophosphate compound and impose a challenge to vector control operation in South Asia (18, 20).

## Recent advances in drugs and vaccines

There is a constant global effort to develop vaccines against dengue virus such as Dengvaxia, LATV, TAK-003, TDEN F17/F19, DPIV TVDV, V180. However, Dengvaxia is the only commercially available vaccine that offers protection against all four dengue serotypes. It is recommended to receive this live attenuated immunization if you have already infected dengue. This decreases the effectiveness of the vaccine, since people who have never experienced the disease are more susceptible to developing severe dengue (21). In the Philippines, there was controversy after more than 733,000 children and 50,000 adult volunteers received the Dengvaxia vaccine regardless of their serostatus in 2017. Mahidol University developed another potential dengue vaccine, TAK-003 or DENVax, and phase I and II clinical trials were conducted in Singapore and Thailand. So far, it has induced sustained antibody responses against all four virus strains, regardless of previous dengue infection. Dengue vaccinations, unlike those used to protect people against COVID-19, took a long time to develop (21, 22).

## Role of COVID-19 lockdown

The increasing case load during COVID-19 period can be theoretically explained in two ways: First, the COVID-19 induced lockdown and restrictions disrupt the pathogen-vector-host relationship, but mobility restrictions lead to increased significant contact with the vector; for example, working from home requires people to spend a considerable amount of time in the neighborhood, which increases the potential risk of human vector contact regardless of the vector concentration of this locality (23). Second, the disruption to regular vector control programs is considered to be the cause of the detrimental impacts of lockdown for dengue transmission. The interruption of adult mosquito-killing practices, such as indoor residual spraying and, where effective, space spraying, is likely to have the most noticeable and rapid effects (24). Furthermore, the similarity of the signs and symptoms causes a delay in diagnosis and treatment, leverage the front-line workers to combat the newly immersed pandemic can lead to neglect of other health programs, which is another contributing factor to the rise in dengue cases during the pandemic. Fear of developing COVID-19 infection leads to a 30–40% reduction in medical appointments in the region, leading to dengue mortality and morbidity (25).

## Policy implications

Although medical attention is focused on the COVID-19 pandemic, it is vital to prioritize vector control efforts in response to this increase in dengue infection. Countries should prioritize digitization of vector-borne disease surveillance programs by establishing mobile-based surveillance systems that enable local and national health authorities. Meanwhile, local governments should focus on educating communities and empowering them to take action to disrupt mosquito life cycles. The concern authority should keep a close check on the number of incidents in tropical and subtropical countries throughout heatwaves since these may raise the potential dengue outbreaks and using weather forecasts to encourage public health protection. Dengue vector control personnel should concentrate their efforts on conserving buildings and other infrastructures to implement sustainable vector control measures. Furthermore, to avoid misdiagnosis and delayed intervention, both dengue and COVID-19 management should be undertaken simultaneously. Immunization should be mandated for these two infectious diseases. However, more studies are also necessary to understand the connection between the two diseases, particularly the phenomenon of cross-reactivity.

Dengue continues to be a neglected disease in South Asia with multifactorial challenges for its management. Current surveillance systems adopted by most countries in South Asia do not provide adequate information for timely management of the DENV. Therefore, an integrated electronic entomological surveillance is warranted to understand the geospatial and temporal distribution of vector populations for targeted interventions. Additionally, the management of dengue necessitates multidisciplinary health interventions encompassing several elements of society. The focus of these actions should be on epidemiological monitoring, quicker and more precise diagnosis, training of health care workers, and community engagement.

## Author contributions

All authors listed have made a substantial, direct, and intellectual contribution to the work and approved it for publication.
